# Mind the gap: Development and validation of an evolutionary mismatched lifestyle scale and its impact on health and wellbeing

**DOI:** 10.1016/j.heliyon.2024.e34997

**Published:** 2024-07-23

**Authors:** Jiaqing O, Trefor Aspden, Andrew G. Thomas, Lei Chang, Moon-Ho Ringo Ho, Norman P. Li, Mark van Vugt

**Affiliations:** a Department of Psychology, Aberystwyth University, United Kingdom; b School of Psychology, Swansea University, United Kingdom; c Department of Psychology, University of Macau, Macao; d School of Social Sciences, Nanyang Technological University, Singapore; e School of Social Sciences, Singapore Management University, Singapore; f Department of Experimental and Applied Psychology, Vrije Universiteit Amsterdam, the Netherlands

**Keywords:** Evolutionary mismatch, Health, Wellbeing, Scale construction, Lifestyle, Environment

## Abstract

Identifying an integrative framework that could appropriately delineate underlying mechanisms and individual risk/protective factors for human health has remained elusive. Evolutionary mismatch theory provides a comprehensive, integrative model for understanding the underlying causes and mechanisms of a wide range of modern health and well-being problems, ranging from obesity to depression. Despite growing interest regarding its importance though, no psychometrically-sound measure of evolutionary mismatch yet exists to facilitate research and intervention. To construct such a scale, aimed at gauging individual differences in the extent to which people's modern lifestyles are mismatched with ancestral conditions, we conducted four studies (a pilot study, followed by 3 main studies, with a final sample of 1901 participants across the main studies). Results from exploratory and confirmatory factor analyses have produced a 36-item evolutionary mismatched lifestyle scale (EMLS) with 7 subdomains of mismatched behaviours (e.g., diet, physical activity, relationships, social media use) that is psychometrically sound. Further, the EMLS is associated with physical, mental and subjective health. We explore the potential of the EMLS as a tool for examining interpersonal and cultural variations in health and wellbeing, while also discussing the limitations of the scale and future directions in relation to further psychometric examinations.

## 1 Introduction

Statistics have shown that most deaths or disabilities recorded from around the globe could typically be attributable to health factors (e.g., Refs. [[1], [2], [3]]), with the development of the majority of them influenced, at least in part, by deleterious lifestyle choices such as having a poor diet, not involving in physical exercise, and more generally, coping inadequately with the pressures of modern life [[4], [5], [6]]. Indeed, studies have found that modifications to one's way of living can have a significant impact in lessening one's likelihood of experiencing adverse physical and mental health issues [6,7]. Despite the undeniable value of such primary preventive efforts in helping to stop health conditions from emerging at the outset however, some challenges do persist.

Pinpointing lifestyle determinants to target (and understanding the fundamental interactive processes at play) in order to prevent health conditions from developing at all appears to be an onerous task to undertake, and many preventive strategies often do not target the actual underlying determinants [8]. Adopting a more unifying approach toward health conditions (rather than looking at each of them separately) could be an effective method to counteract such challenges, as is utilising an overarching framework to clarify core determinants [8]. Such an all-embracing, yet parsimonious focus could be instrumental in providing that coherent glue (and “story”) which could help to link all the commonly-identified health-related lifestyle factors (e.g., social support, physical activity, diet and many others) together. While each of these factors have been widely examined and theorised about, there is a need for a conceptual framework that could provide a consistent, all-inclusive rationale for why they could be important for one's health.

### 1.1 Evolutionary mismatch theory

To this end, viewing the impact of modern lifestyles on health and wellbeing through the lens of evolutionary mismatch theory offers a promising approach to achieving these two objectives (e.g., Refs. [9,10]). It is a comprehensive and yet parsimonious perspective that argues that many contemporary health and well-being issues are due to a dissimilarity between the lifestyles of humans in the ancestral environments in which they have evolved from -- which have shaped current psychological and physical mechanisms/traits because they have solved a range of adaptive challenges encountered by early humans [11,12] -- and the substantially evolutionarily unfamiliar and environmentally novel world that modern humans currently inhabit. Such outcomes are believed to have occurred because the rate of change that characterises modern life has far surpassed that of humans’ ability to transform through the gradual process of evolution by natural selection, creating the potential for evolutionary mismatch (see Refs. [[13], [14], [15], [16]]; for an overview of this formulation). A classic mismatch example is the higher risk among (some) modern humans in terms of developing metabolic syndrome due to a combination of a sedentary lifestyle and a diet made up typically of processed high-fat food that is packed with salt/sugar, which are in stark contrast to the largely minimally-processed (if at all) food and active lifestyle of our prehistoric ancestors [13].

There is a notable intensification of attention in the scientific community on evolutionary mismatch theory and its utility for understanding the human condition in recent years. Evolutionary mismatch theory has been used to explain the deeper causes of an assortment of health complications, including osteoarthritis [17]; premenstrual syndrome [18]; abnormal immune responses to certain food [19]; various types of cancer [20]; autism [21]; attention deficit hyperactivity disorder [22]; insomnia symptoms [23]; and depression [[24], [25]]. However, there is a lack of empirical research that has directly tested the contributing role of evolutionary mismatch on behaviours that are predictive of one's personal health and subjective wellbeing (for exceptions, see, e.g., Refs. [26,27]). One key reason for the discrepancy between theory and investigation could be the lack of a convenient way to comprehensively measure a broad concept like evolutionary mismatch and how it differs between individuals, subgroups of individuals and possibly even societies.

### 1.2 How evolutionary psychology can offer ideas

**Fig. 1:**
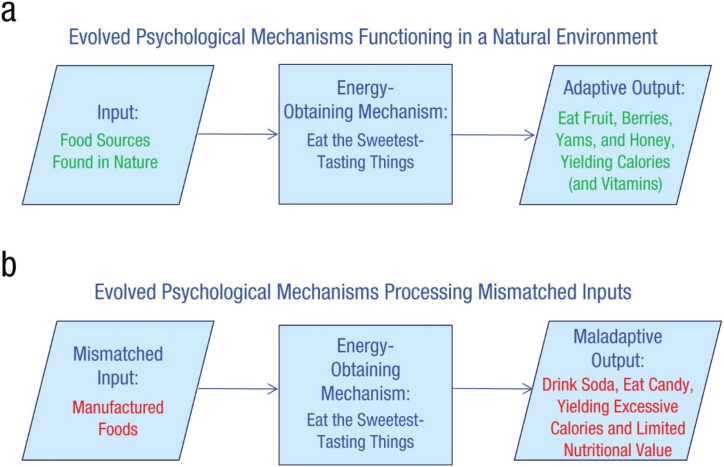
“Schematic showing (a) how an evolved psychological mechanism functions in a natural environment and (b) how the same psychological mechanism functions in a modern context. Although the same decision rule is followed in each case, different inputs lead the mechanism to produce different outputs. As a result, mechanisms that were evolutionarily beneficial can be maladaptive in the modern world”. Reprinted with permission from Li, N. P., van Vugt, M., & Colarelli, S. M. (2018). The Evolutionary Mismatch Hypothesis: Implications for Psychological Science. *Current Directions in Psychological Science.* Copyright 2018, Association for Psychological Science.

Evolutionary psychology offers a potential avenue for an empirical assessment of evolutionary mismatch [28]. Evolutionary psychology assumes that the human mind has evolved to solve various distinct problems relevant to human survival and reproduction by means of providing domain-specific solutions, called evolved psychological mechanisms or psychological adaptations [11]. These are if-then decision rules (mental algorithms) that are activated when humans experience challenges in different life domains such as in the context of food intake, physical activity, work and romantic relationships. These adaptations are fine-tuned to ancestral environmental conditions, but when these conditions rapidly change however – such as with the current abundance of cheap, processed foods in supermarkets among many other evolutionarily-unfamiliar things – they may backfire by encouraging people to make lifestyle choices that may be immediately gratifying while negatively impacting their health and well-being in the long run. More specifically, consider how mismatch can be applicable to various lifestyle choices (e.g., diet), where different environmental stimuli (e.g., food that is available in a natural setting vs. evolutionarily unfamiliar processed food that is available in the supermarkets) could both potentially activate the same evolved mechanism in ways that might produce either positive health behaviours (e.g., eating wholesome natural food) or otherwise (e.g., eating unhealthy processed food) [15]. While the same purposeful approach – “eat the sweetest-tasting things” you can find in order to give you energy for sustenance [15, p. 40] -- is adopted in both situations, distinct environmental stimuli could bring about divergent outcomes even if the same evolved mechanism is being initiated [15] (see Fig. 1). In short, mechanisms that have evolved to help people survive and/or to reproduce could nonetheless be counterproductive in the current context due to evolutionary mismatch [15].

Based on the evolutionary psychology literature we can identify a number of domains in which evolutionary mismatched conditions are likely for humans living in large, modern, complex, industrialised and digitised societies. The genus Homo emerged over 2.5 million years ago in Africa and one lineage evolved over time into what we are, e.g., Homo Sapiens, or anatomically modern humans (AMH). About 50,000 years ago, small groups of AMH moved out of Africa in different migration waves and, in a relatively short time span, occupied the rest of the planet. An undisputed claim from evolutionary psychology is that the psychological adaptations that characterise modern humans (e.g., language, intelligence, food and mate preferences) – just like the physical adaptations (e.g., bipedalism) – were already in place before AMH moved out of Africa. Yet the world that AMH currently inhabits looks very different from our ancestral world, especially for those of us living in large, modern, complex societies.

Humans are the ultimate niche constructors and due to their cultural and intellectual capacities have significantly altered their physical and social environment. A case in point is the agricultural revolution that happened about 12,000 years ago, which has significantly changed the lifestyle of humans in terms of what they eat, how they live and how they work [29]. In addition, the industrial and the recent digital revolution have also produced significant changes in common behavioural patterns, including in the way we communicate with each other [30]. This creates the potential for evolutionary mismatches as these new behavioural patterns and lifestyle choices may not align very well with our evolved psychological mechanisms.

Beyond a substantial change to our diets, our level of physical activity has also changed dramatically [31]. In ancestral environments, humans needed to engage in physical activity – as hunters and gatherers – in order to obtain the foods necessary for their survival and that of their families. In a modern world where foods are easily accessible, people generally lead a more sedentary lifestyle, which has contributed to all kinds of health and well-being problems (e.g., obesity and cardiovascular disease). Another candidate for mismatch is the home environment in the modern world. Ancestral humans spend most of their time in natural environments as opposed to the urban, human man-made environments and they had plenty of access to nature wherever they lived. Indeed, several studies have reported a link between having access to nature and better physical and mental health (e.g., Refs. [32,33]).

Our ancestors also lived and worked for the most part in small bands composed largely of kin [34]. They spent most of the time with people they knew intimately and the tight social networks they had would have offered a lot of social support. As the majority of people nowadays live and work in highly urbanised environments, they would likely spend most of their time away from relatives and be surrounded by people they barely know in various comparatively less cohesive groups [35], potentially leading to increased feelings of isolation and loneliness. Accordingly, research has also established a link between feelings of loneliness and depression [36].

Similarly, mismatches might occur in the context of romantic relationships [37]. Mate choice was arguably quite restricted in ancestral environments due to constraints on group size and the enormous influence of family on mate choices, and as a result, mate acquisition skills were probably not much needed then [38]. The evolutionarily-recent relative freedom to search for one's own mate – based on personal preferences and experiences -- could potentially affect one's well-being negatively if people have not had the chance to develop mate acquisition skills [39]. A similar impact on one's well-being due to the modern-day emergence and widespread usage of (digital) social media tools could also be observed. Indeed, research findings have suggested that frequent comparisons on social media (where people could easily curate the type of lifestyle or physical appearance they want others to believe they have, even if it is not entirely real) were linked to poorer mental health, in the form of greater symptoms of depression [40] and greater risk of pathological eating [41].

Note that there may be additional behavioural domains that could potentially be evolutionarily-mismatched in the modern world, such as modern parenting skills for instance, which might impact human wellbeing in various ways. However, the discussion for this paper is focused on individual lifestyle choices which we know are influential in affecting personal health and well-being based on the empirical literature, and have probably undergone the most amount of changes since ancestral times. Several of the above-mentioned lifestyle domains have already been linked to and analysed through the lens of evolutionary mismatch theory. However, they have not been fully investigated empirically in the absence of a methodology to gauge individual differences in various relevant and potentially mismatched lifestyle domains. The availability of such an assessment tool would facilitate more research in this emerging, important area of inquiry of human health by offering a quantitative, easy-to-score measure of the discrepancy between one's modern lifestyle and that of their prehistoric ancestors. Integrating these different elements into one general scale with different sub-dimensions also has the added value of determining which of these mismatched lifestyle domains are most important in undermining one's health and well-being.

### 1.3 Purpose of this project

In view of the growing recognition of evolutionary mismatch theory as a broad-ranging concept to understanding and exploring the origins of health problems, the main goal of this paper is to develop a scale to measure the extent to which people's current lifestyles are mismatched with what we know from the anthropological literature about ancestral human lifestyle. This measure, which we shall refer to as the evolutionary mismatched lifestyle scale or, EMLS in short, could provide an efficient way of capturing evolutionary mismatch across a number of relevant life domains. It would help us broaden our understanding of those health issues that have already been conceived to be associated with specific forms of mismatch and would allow for exploratory testing of the role of mismatch in health domains not previously considered. Hence, this project aims to psychometrically construct, to the best of our knowledge, the first-ever empirically-evaluated measure of evolutionary mismatched lifestyle behaviours. This will be done via a pilot study and three main exploratory studies on the EMLS. The pilot study will first be conducted to ensure that the proposed EMLS are clear and relevant; and this will be followed by an identification of the most psychometrically-relevant EMLS via an exploratory factor analysis (EFA) in study 1 and then via a confirmatory factor analysis (CFA) that will be used to refine the scale in study 2. CFA will be used in study 3 to confirm the structural validity of the scale which will be developed through the empirical work in studies 1 and 2, and the final scale will additionally be tested for its reliability (test of internal consistency in main study 3), discriminant (e.g., in comparison with scales of urbanicity and tolerance of ambiguity) and convergent validity (e.g., in comparison with a healthy lifestyle scale). We will also measure predictive validity in terms of the scale's ability to predict outcomes such as mental wellbeing, physical health issues, and overall health cross-sectionally in study 3.

## 2 Methods

The generation of proposed items for the EMLS at the outset was based on the large body of indirect empirical research that could be viewed through the lens of evolutionary mismatch, coupled with feedback and the contribution of ideas from several experts in evolutionary behavioural sciences in relation to the potential types of items/domains that could be relevant in light of the evolutionary mismatch framework (some took part at a later stage of the process). Eighty-nine items were proposed as a result, with items ranging from diet-related ones to questions about one's social support network at home and work. These domains are understood to be largely in line with existing research on some of the commonly identified health-related factors in health and clinical psychology (e.g., Refs. [[42], [43], [44]]). Ethical approval for the pilot study and all the three main studies were obtained from Aberystwyth University's Department of Psychology ethics committee (AU online ethics number: 10720) on the March 20, 2019, prior to the start of the project. Additional ethical approval was subsequently also obtained for part of the research work from Swansea University's Department of Psychology Ethics Committee (ethics number: 1539) on the October 14, 2019. Informed consent was also obtained from all participants – they were asked to provide their consent by selecting “I agree” after reading the Participant Information and Informed Consent section of the online questionnaire in order to proceed with each study. A pilot study was initially conducted to evaluate the proposed items in terms of clarity and suitability. This was then followed up by 3 main studies that served to identify the most appropriate items and/or to establish the psychometric properties of the scale. The experts were then given a chance to review the findings and to make appropriate suggestions and recommendations.

### 2.1 Pilot study: procedure

Participants were recruited via Prolific (www.prolific.ac) and were given an online questionnaire to complete in return for a small monetary incentive. To ensure high quality data, only individuals who had completed at least five previous studies on Prolific and had at least 99 % of their previous contributions endorsed by other researchers on the platform, were eligible to be recruited to participate in this study. Participants also had to be at least 18 years old and be based in the UK. Those who did not meet these criteria were automatically excluded from participating via the platform. In order to enhance participants' attentiveness to the survey, they were presented with an adapted version of Bayram's [45] empirically-supported statement highlighting the significance of paying careful consideration to the questions before the start of the questionnaire.

### 2.2 Pilot study: participants

Fifteen eligible individuals completed the study, with 12 (80 %) of them having indicated “female” as their biological sex. Participants ranged from 21 to 61 years old and have an approximate mean age of 35.

### 2.3 Pilot study: measure

The questionnaire for the pilot study consisted primarily of the list of 89 preliminary items that was developed by the research team for the purposes of this project as described above. Following a brief explanation of what evolutionary mismatch was about,^1^ participants were asked to indicate if each item was clear to them on a 7-point Likert scale which ranged from “very unclear” to “very clear” and whether they agreed that the items could sufficiently assess the extent to which one's current environment was “natural” or “unnatural” via response options that varied from 1) “strongly disagree” to 7) “strongly agree”. Items that scored under 6 out of 7 on average for at least one of these aspects were further examined with a view to make potential modifications where appropriate. Participants were also provided with the option to suggest any other possible items they believed might fit the scale.

### 2.4 Pilot study: results

In terms of clarity, 81 out of 89 (or 91.01 %) of the items were regarded as appropriate (e.g., they were assessed by the participants to have at least 6 out of 7 in terms of clarity on average). The remaining eight items were modified to enhance their clarity to participants. In contrast, 88 out of 89 (or 98.88 % of the) items, on average, were surprisingly not deemed by the participants to adequately reflect what is “natural” or “unnatural” in one's environment. Given that participants may not have understood this question very well (perhaps because of the complexity and abstractness of the concept), and that the items were jointly developed with experts who have knowledge about evolutionary concepts (who have provided useful inputs regarding the kinds of potential domains/items that were potentially relevant to the EMLS) and were based on existing scientific knowledge of evolutionary mismatch, there were reasons to believe that the notion of evolutionary mismatch might have been too abstract for participants (if they do not have any background in the field) to grasp (despite the definition provided) especially given the excessively huge number of items that were rated as being inadequate. Hence, the decision was made to use only clarity ratings when assessing the items initially and when it is time to make further adjustments in light of the factor analyses in the subsequent studies. Participants also suggested five new ideas for items. Four of these were regarded as relevant by the research team and were incorporated into the main measure.

### 2.5 Study 1

Study 1 was designed to psychometrically assess and refine the proposed items for inclusion in an EMLS scale.

### 2.6 Study 1: Procedure

The recruitment and study procedures and the inclusion/exclusion criteria were identical to those of the pilot study, except those individuals were additionally excluded from participating in this study if they were already involved with the pilot study.

### 2.7 Study 1: Participants

Eight hundred and one sets of complete data were obtained, but two participants were believed to have provided two sets of data and so the decision was made to only keep the primary data (which was assumed to be more accurate). Therefore, (primary) data from a total of seven hundred and ninety nine participants were used for the final analyses. 40.8 % (326) of the sample were men and 59.2 % (473) were women. The approximate mean age of the sample, which ranged between 18 and 87 years old, was 37. Annual income (before tax) of participants varied over a wide range: 23.4 % (187) of participants reported an income under £10,000; 21.5 % (172) reported an income between £10,000 and £19,999; 24.9 % (199) reported an income between £20,000 and £29,999; 15.5 % (124) reported an income between £30,000 and £39,999; 7.6 % (61) reported an income between £40,000 and £49,999; 3.9 % (31) reported an income between £50,000 and £59,999; and 3.1 % (25) reported an income of £60,000 or over. 85.7 % of the sample were residents of England, and 8 % were based in Scotland; participants who were located in Wales and Northern Ireland made up the remaining 5.5 % and 0.8 % of the sample respectively.

### 2.8 Study 1: Measures

A modified list of 93 items assessing the concept of evolutionary mismatch, based on the findings in the pilot study (as described earlier), was utilised. Response options ranged from 1 = “strongly disagree” to 7 = “strongly agree”. Higher scores denote greater evolutionarily-mismatched experiences. As a suggestion, if any subscale of the EMLS contains missing data, the subscale can be computed using the mean of the scores of other items within the subscale that do not contain missing data, assuming at least 75 % of the data for these items are non-missing.

### 2.9 Study 1: Data screening

Multivariate normality of the data was assessed using the Mahalanobis distance statistic and the associated chi-square p-value. To screen for multivariate outliers, we sorted Mahalanobis distance values in ascending order within the dataset, to screen for sudden jumps in Mahealani's distance among the higher values.

The majority of participants (82 %) had multivariate normal data (*p* ≥ 00.05), with 18 % showing some deviation from multivariate normality. Three participants could be regarded as borderline outliers with a jump in Mahalanobis value from the next lowest value. We chose to err on the side of inclusion, retaining all participants, including outliers in the analysis.

### 2.10 Study 1: Analyses

EFA conducted in SPSS 25 was used to explore whether coherent dimensions could be formed from the EMLS items, and to refine the number of items. The EFA was conducted in accordance with commonly used guidelines [46], using principal axis factoring and oblimin (a form of oblique) rotation. In determining the dimensionality of the scale, we used a combination of eigenvalues (retaining factors with eigenvalues above 1) and scree plots (retaining eigenvalues that occurred above the “bend”). In determining items to retain, we examined the pattern matrix and aimed to retain items with factor loadings >0.4. We examined internal consistency of all scales using Cronbach's alpha, with values between 0.7 and 0.95 being regarded as good. In addition to the primary analysis which has included outliers, we have also performed a sensitivity analysis by repeating the same EFA after excluding the multivariate outliers.

### 2.11 Study 1: Results and discussion

Based on eigenvalues and the scree plot, a solution consisting of six factors was adopted. 62 of the original 93 items were retained. This resulted in the following scales: *Social Media use and Vanity* (15 items); *Relationships with Family, Friends and Colleagues* (9 items); *Neighbourhood and Built Environment* (12 items); *Romantic Relationships and Substance Use* (10 items); *Exercise and Activity* (9 items); and *Diet* (7 items). All the resulting scales demonstrated good internal consistency, with Cronbach's alpha of the scales ranging from 0.73 to 0.82. A sensitivity analysis, by means of the same EFA with outliers removed, has yielded the same six scales. The Cronbach's alpha values for the scales, with outliers removed, fell within a comparable range, ranging from 0.72 to 0.82. These results have provided a tentative overview of the potential kind of relevant items and subdomains of the EMLS.

### 2.12 Study 2

Study 2 was designed to ascertain the factor structure of the proposed EMLS.

### 2.13 Study 2: Procedure

The recruitment and study procedures and the inclusion/exclusion criteria were identical to those of study 1, except those individuals were also excluded if they have previously participated in either the pilot study or study 1.

### 2.14 Study 2: Participants

Due to a technical issue, some participants were believed to have redone the study, presumably resulting in 22 additional sets of data from the same individuals. Assuming that the primary data from these participants were more accurate, data from the second attempt were removed from all analyses and the final sample of participants who completed the study amounted to 550 participants. In terms of biological sex, 397 (72.2 %) were females and 153 participants (27.8 %) were males. The sample varied from 18 to 75 years old, with an approximate mean age of 35 (age data from one participant appeared unfeasible and so was excluded from all analyses relating to age). Annual income (before tax) of participants spanned across several income ranges: specifically, 31.1 % (171) of participants had an income that was under £10,000; 21.1 % (116) had an income between £10,000 and £19,999; 21.8 % (120) had an income between £20,000 and £29,999; 14.7 % (81) had an income between £30,000 and £39,999; 5.8 % (32) had an income between £40,000 and £49,999; 2.4 % (13) had an income between £50,000 and £59,999; and 3.1 % (17) had an income of £60,000 or over. 85.1 % of these participants were located in England, while 9.1 % resided in Scotland. 4.9 % were residents in Wales and another 0.9 % were inhabitants of Northern Ireland.

### 2.15 Main study 2: Measures

A shortened, 62-item version of the EMLS, based on the findings in main study 1, was adopted for this study.

#### 2.15.1 Study 2: Data screening

Multivariate normality of the data was assessed using Mahalanobis distance statistic and the associated chi-square p-value. We screened for multivariate outliers by sorting Mahalanobis distance values in ascending order, to screen for sudden jumps in Mahalanobis distance among the higher values.

86 % of participants had multivariate normal data (*p* ≥ 00.05), with 14 % showing some deviation from multivariate normality. Examining the Mahalanobis distance indicated that no participants were multivariate outliers, and all participants were therefore retained in the analysis.

### 2.16 Main study 2: Analyses

CFA conducted in AMOS 26 was used to confirm the dimensionality of the scales derived from study 1. Model fit was determined using a combination of Comparative Fit Index (CFI) and Standardised Root Mean Square Residual (SRMR) [47], with values of CFI >0.90 and SRMR ≤0.09 being taken to indicate adequate model fit. To account for similarities between items other than those caused by the factor, error terms of two or more items within a factor were allowed to correlate when 1) high modification indices suggested this to be necessary and 2) there were also strong theoretical grounds (e.g. similar item wordings) to justify this [48]. Based on the results of the CFA, adjustments were made to the dimensionality of the scales (e.g., splitting scales where this was statistically and theoretically valid). The number of items was also further refined, with item removal based on a combination of statistical (e.g. factor loading, improvement in model fit) and theoretical (e.g. how well the item reflects the overall factor) reasons.

### 2.17 Main study 2: Results and discussion

The scales derived in Study 1 were modified further based on the results of the confirmatory factor analysis. The scale *Social Media Use and Vanity* was split into two separate scales, one measuring *Social Media Use* and the other *Vanity*, based on conceptual differences and an improvement in model fit. Items on substance use were removed from the *Romantic Relationship and Substance Use* scale, which was relabelled *Romantic Relationships*, based on conceptual differences and poor item-factor loadings. And from the *Built Environment and Neighbourhood* scale, items on the workplace environment were removed, as they displayed only a weak loading onto this scale, which was composed mainly of items about the neighbourhood (home) environment.

The resulting model, which comprised 7 scales consisting of a total of 36 items, demonstrated an adequate fit to the measurement model: χ^2^ (567) = 1224.18, CFI = 0.91, SRMR = 0.07. RMSEA was 0.046 (90 % CI 0.42–0.49), also indicating adequate fit to the measurement model.

The scales in this final model were: *Social Media Use* (6 items), *Vanity* (3 items), *Social* Support (7 items), *Home Environment* (5 items), *Romantic and Sexual Relationships* (6 items), *Physical Activity* (4 items), and *Diet* (5 items). These subscales closely tap into the distinct life-domains identified by various evolutionary psychologists as being affected by mismatch.

### 2.18 Study 3

Study 3 was designed to assess the reliability (e.g., internal consistency) and validity (e.g., structural, predictive, and convergent/divergent validities) of the proposed EMLS scale.

### 2.19 Study 3: Procedure

The recruitment and study procedures and the inclusion/exclusion criteria were likewise identical to those stipulated in Study 1, except those individuals were also excluded from participating if they had already done so in any previous study of this project.

### 2.20 Study 3: Participants

As there were two sets of data that could not be reliably determined to have come from 2 different participants, only the primary set of data was retained (as per the same approach in the preceding studies). One set of test data was also removed, which resulted in a final sample of 552 individuals who have completed the study. After excluding data from one additional individual from all age-related analyses due to an unfeasible reported age, the final sample had an approximate mean age of 37, ranging from 18 to 74 years old. 361 participants (65.4 %) indicated they were biological females, and 191 others (34.6 %) were biological males. An assortment of different annual income (before tax) levels was likewise observed among this group of participants: in particular, 27.9 % (154) of them reported that they were making under £10,000; 23.4 % (129) reportedly made between £10,000 and £19,999; 24.5 % (135) made between £20,000 and £29,999; 12.5 % (69) made between £30,000 and £39,999; 5.4 % (30) made between £40,000 and £49,999; 2.7 % (15) made between £50,000 and £59,999; and 3.6 % (20) made £60,000 or over. Most participants (83.7 %) were residing in England, with much smaller numbers of them reportedly located in other regions such as Scotland (9.2 %), Wales (6 %) and Northern Ireland (1.1 %) respectively.

### 2.21 Study 3: Measures

#### 2.21.1 Evolutionary mismatched lifestyle scale

A final, 36-item measure of ELMS (see Appendix B), based on findings in main study 2, was used for this study.

### 2.22 Measures of health

*Physical Health Issues.* Schat, Kelloway and Desmarais' [49] 14-item Physical Health Questionnaire, which measures problems with physical health such as issues with sleep, digestion, and infections, was used to assess participants' physical wellbeing. Participants were provided with a range of response options that varied from “not at all” to “all of the time” for all the items in this study [49, p. 375]. All items (except for tallies for an item on one's experiences of a calm sleep which have to be transformed first as they were reverse-scored) were summed to provide an overall rating of one's physical health issues.

*Mental Wellbeing.* Mental wellbeing of each participant was assessed using Tennant and colleagues’ [50] authoritative 14-item Warwick-Edinburgh Mental Well-being Scale (WEMWBS).^2^ WEMWBS was developed by the Universities of Warwick, Edinburgh and Leeds in conjunction with NHS Health Scotland. Permission has been obtained from the developers prior to its utilisation in this study. Participants were asked to rate their psychological experiences via 5 response options that ranged from “none of the time” to “all of the time” [50, p. 3].

*Overall Health.* Overall health of the participants was evaluated using Mossey and Shapiro's [51] widely used one item measure of subjective health. Participants were given a range of 5 options to select from that varied from “excellent” to “bad” in relation to the question “for your age would you say, in general, your health is” [51, p. 801]. Scores for items were reverse-coded in this study such that a higher value would equate to better overall health.

### 2.23 Validation measures

*Extent of Urbanicity.* An adapted, shortened version of Novak, Allender, Scarborough, and West's [52] urbanicity scale was utilised to assess the extent to which participants' living environments are urbanised. It comprised a variety of questions that assesses a diverse range of indicators of urbanicity, including education, healthcare, amenities, technology use, nature of roads, the extent of agricultural work and the number of people in the area [52]. After taking into account the reverse-scoring of items relating to the nature of roads and the extent of agricultural work, all items are then summed to provide an overall tally of the extent of how urbanised each participant's living environment was. This measure serves as an indication of whether the EMLS has discriminant validity. Although an urbanised environment could arguably be more likely to be evolutionarily-mismatched than its rural counterpart, the fact that the EMLS is focused on the deleterious aspects of evolutionary mismatch would mean that it could conceptually be fairly different from the extent of urbanicity scale as the latter would likely also encompass health-benefitting evolutionarily-mismatched aspects of urbanisation (e.g., the presence of a wide array of healthcare facilities). In addition, the belief that evolutionarily-mismatched factors are not restricted only to the modern, urban environment (but are also likely apparent in relatively evolutionarily-recent agricultural settings as well) would also suggest that the EMLS and the degree of urbanicity should be regarded as fairly dissimilar entities.

*Tolerance of Ambiguity.* Participants' tolerance for ambiguity will be measured by Herman, Stevens, Bird, Mendenhall, and Oddou's [53] 12-item scale that was designed to assess this construct. Each participant was presented with response options that varied between “1 = strongly disagree” and “5 = strongly agree” [53, p. 64]. Tallies for 7 items must be transformed first as they were reversed scored, before the ratings were all totalled. This scale is similarly utilised to test if the EMLS has discriminant validity as stimuli/experiences may arguably generate discomfort from being unclear in their function or form but are not evolutionarily-mismatched environmental factors (nor would they necessarily generate comparable subsequent consequences) per se. For instance, even if one is highly comfortable with an evolutionarily-mismatched situation, such as being exposed to a lot of strangers in an environment for instance, they might still experience negative health consequences as a result of it in accordance with the evolutionary mismatch framework.

*Healthy Lifestyle.* A slightly-adapted 11-item measure of lifestyle by Gil, Gracia, and Sanchez’ [54] was used to investigate one's level of engagement to a healthy lifestyle. Each item can be answered by choosing one of 7 response options that varied from “strongly disagree” to “strongly agree” [54, p. 224]. All items were summed to provide a total score in relation to the extent of one's adoption of a healthy lifestyle. This measure of healthy lifestyle would be adopted to examine if the EMLS has convergent validity as the EMLS is similarly designed to assess if one's way of life and their environmental exposure could affect one's health and wellbeing.

#### 2.23.1 Study 3: Data screening

Multivariate normality of the data was assessed using Mahalanobis distance statistic and associated chi-square p-value. To screen for multivariate outliers, we sorted Mahalanobis distance values in ascending order within the dataset, looking for sudden jumps in Mahealani's distance among the higher values.

84 % of participants had multivariate normal data (*p* ≥ 00.05). Examining the Mahalanobis distance indicated that five participants could potentially be regarded as multivariate outliers, indicated by a large jump in Mahalanobis distance from the next lowest value. We decided to err on the side of inclusivity, and therefore all participants were retained in the analysis.

### 2.24 Study 3: Analyses

#### 2.24.1 Calculation of EMLS scales

We calculated a scale score for each of the EMLS subscales by taking the item mean of the subscale's constituent items. To calculate an overall scale score for the EMLS, we calculated the mean of the 7 subscales, to provide an EMLS total score. This method of calculating the mean of subscales was chosen to give equal weighting to the separate subdomains (as using the alternative item mean would give greater weighting to subscales containing more items).

#### 2.24.2 Internal consistency and structural validity

Internal consistency reliability of the EMLS and each of its subscales were assessed using Cronbach's Alpha. To confirm the structural validity of the EMLS, CFA was conducted to confirm model fit (with values of CFI ≥0.90 and SRMR ≤0.09 taken to indicate adequate model fit). To ensure the reliability of our results after eliminating outliers, we have also performed a sensitivity analysis by running the CFA without outliers to assess the model's fit. Correlation between subscales was also examined to confirm the structural validity of the scale. Each subscale of the EMLS would expectedly correlate with other subscales at a low to moderate level, reflecting the multidimensional nature of the EMLS, and that each individual subscale would correlate with the overall EMLS at a moderate to strong level (for determining correlation magnitude, significant correlations up to 0.3 were regarded as weak, correlations from 0.31 through 0.6 were regarded as moderate, and correlations above 0.6 were regarded as strong).

#### 2.24.3 Predictive validity

We anticipated that a mismatch between one's evolved psychological/physical mechanisms and the novel environmental conditions would be associated with a reduction in wellbeing and poorer health outcomes. To assess predictive validity of the EMLS we therefore looked at the correlation of the EMLS total with mental wellbeing (assessed with the WEMWBS), negative physical health (assessed through the Physical Health Issues scale), and overall health (assessed with the single item measuring overall health). We hypothesised that the EMLS would be positively associated with physical health issues and negatively associated with mental wellbeing and overall health. We also looked at the R^2^ value from each of these correlations, to estimate the proportion of variance in physical health, mental wellbeing, and overall health that can be explained by the EMLS.

#### 2.24.4 Convergent and divergent validity

Convergent validity of the EMLS total was tested through correlation with the adapted Lifestyle Choices Questionnaire, the Extent of Urbanicity scale and the Tolerance of Ambiguity scale. In line with Hubley's [55] guidelines regarding validity assessment, we expected the EMLS to demonstrate convergent validity (e.g., by correlating significantly and negatively with the adapted Lifestyle Choices Questionnaire) and divergent validity -- by demonstrating that the EMLS has considerably weaker correlation coefficients in relation to both the Extent of Urbanicity scale and the Tolerance of Ambiguity scale.

### 2.25 Study 3: Results and discussion

#### 2.25.1 Internal consistency

All subscales of the EMLS demonstrated a good (or very close to good) level of internal consistency: Cronbach's alpha for *Social Media Use* was 0.76, *Vanity* was 0.69, *Social* Support was 0.84, *Home Environment* was 0.82, *Romantic Relationships* was 0.71, *Physical Activity* was 0.83, and *Diet* was 0.78. Cronbach's alpha of the EMLS total scale was 0.66. For the validation scales Cronbach's alpha for the *Physical Health Issues* questionnaire was 0.78, WEMWBS was 0.91, *Lifestyle Choices* was 0.76, and Tolerance of Ambiguity was 0.70.

#### 2.25.2 Structural validity

Confirmatory factor analysis using the study 3 data confirmed the results from the study 2 analysis, with the model demonstrating an adequate fit to the measurement model (X^2^ (567) = 1275.2, CFI = 0.91, SMRM = 0.07). RMSEA was 0.048 (90 % CI 0.044–0.051), also indicating adequate fit to the measurement model. A sensitivity analysis, conducted using the same CFA with the outliers removed, produced comparable fit indices, with CFI = 0.91, SRMR = 0.07, and RMSEA = 0.048. All of the subscales of the EMLS correlated with all other subscales at a weak to moderate level (Table 1), with the following four exceptions: the *Social Media Use* subscale did not correlate with the *Social* Support or *Romantic and Sexual Relationships* subscales, and the *Vanity* subscale did not correlate with *Social* Support or *Physical Activity* subscales. All EMLS subscales correlated with the overall EMLS score at a moderate to strong level.

**Table 1:** Correlations between subscales of the EMLS, *Study 3*.

	Variable	*M*	*SD*	1	2	3	4	5	6	7	8
1	Social Media Use	3.6	1.31	1							
2	Vanity	2.38	1.25	0.49**	1						
3	Social Support	3.98	1.4	0.06	−0.01	1					
4	Home Environment	4.44	1.41	0.26**	0.15**	0.38**	1				
5	Romantic and Sexual Relationships	2.92	1.18	0.08	0.14**	0.24**	0.23**	1			
6	Physical Activity	4.01	1.59	0.21**	0	0.26**	0.25**	0.13**	1		
7	Diet	3.26	1.33	0.37**	0.12**	0.29**	0.27**	0.13**	0.43**	1	
8	EMLS Total	3.51	0.78	0.60**	0.45**	0.57**	0.64**	0.46**	0.61**	0.66**	1

*p < 0.05 level: **p < 0.01 level.

#### 2.25.3 Predictive validity

Findings demonstrated the hypothesised moderate negative correlation between the EMLS and the WEMWBS and the overall health item, with R^2^ indicating that the EMLS can account for 28 % of the variance in the WEMWBS and 10 % of the variance in the overall health item. Findings also demonstrated the hypothesised positive correlation with the Physical Health Issues scale, with R^2^ indicating that the EMLS total can account for 12 % of the variance in the Physical Health Issues scale.

#### 2.25.4 Convergent and divergent validity

Results demonstrated the hypothesised negative correlation between the EMLS total score and the Lifestyle Choices questionnaire. The correlation coefficient of the EMLS total -lifestyle choices relationship (*r* = −0.52) is considerably greater than that of the EMLS-Tolerance of Ambiguity relationship (*r* = −0.26), while results have shown that the EMLS is unrelated to the extent of urbanicity scale (*r* = 0.02) (see Table 2).

**Table 2:** Correlations between the EMLS and validation variables.

	*M*	*SD*	EMLS Total	Social Media Use	Vanity	Social Support	Home Environment	Romantic and Sexual Relationships	Physical Activity	Diet
Physical Health Issues	41.08	12.41	0.34**	0.36**	0.26**	0.19**	0.11*	0.06	0.18**	0.19**
Positive Mental Wellbeing	44.46	8.83	−0.53**	−0.24**	−0.10*	−0.52**	−0.31**	−0.28**	−0.35**	−0.30**
Overall Health	3.70	0.82	−0.32**	−0.11**	0.00	−0.22**	−0.12**	−0.11**	−0.41**	−0.24**
Extent of Urbanisation	85.45	6.10	0.02	−0.05	−0.03	0.02	0.24**	0.02	−0.05	−0.08
Lifestyle Choices	44.94	10.20	−0.52**	−0.20**	−0.01	−0.26**	−0.21**	−0.15**	−0.50**	−0.68**
Tolerance of Ambiguity	36.72	6.16	−0.26**	−0.12**	−0.03	−0.31**	−0.16**	−0.04	−0.17**	−0.18**

*p < 0.05 level; **p < 0.01 level.

### 2.26 General discussion

All in all, the present findings have provided empirical support for a first-of-its kind, psychometrically sound measure of evolutionary mismatch that taps into individual differences in the extent to which people's lifestyles and environmental conditions differ from those in which humans evolved (see Table 3 for an overview of the various domains that were covered by the scale). The scale is based on the well-established idea from evolutionary biology, medicine and psychology that mismatches between our modern lifestyles and that of our ancestors', can, in part, account for a wide range of deleterious outcomes for individuals [9,12,15,16]. Importantly, our results have demonstrated that the EMLS, as predicted, is linked to both physical and psychological wellbeing and one's general health as a whole, as assessed using well-established and reliable measures. The EMLS provides an integrated approach (spanning several key areas of one's way of life) to assessing health and wellbeing that goes beyond existing means of evaluation that only focus on individual lifestyle domains.

**Table 3:** EMLS Subscales and their Respective Items.

Social media use	
	Send a lot of texts every day
	Compare life with those of people on SM
	Compare appearance with those of people on SM
	A lot of “friends” on SM who are mere acquaintances
	Friends generally use devices most of time
	Time on Facebook, Twitter, and social media
**Vanity**	
	A lot of beauty/grooming products
	Look at self in mirror
	Look at images and videos of myself
**Social Support**	
	Not close to people in work/place of study
	Very limited interaction with relatives
	Emotionally connected to very few family members
	Few family members I could count on
	Few close friends who I could count on
	Few close friends I can talk to about problems
	Not listened to at my workplace
**Home environment**	
	Live in area densely populated with strangers
	Exposed to very little nature in home environment
	Limited interaction with people in neighbourhood
	Do not know most people in neighbourhood
	Do not know most immediate neighbours
**Romantic/sexual relationships**	
	Large part of life living alone
	Have to meet new people and flirt with them
	Using apps and dating sites to hook up and have sex
	Have to rely on own efforts romantic relationships
	Usually sleep alone
	Using apps of dating sites to find romantic partners
**Physical Activity**	
	Not physically active
	Do not exercise regularly
	Large part of leisure time sat on chair
	Less than half hour exercise per day
**Diet**	
	Processed meat and vegetables
	Sweetened beverages
	Food high in fat content
	Eat a lot of sugary stuff
	Do not eat much fruit, vegetables, and nuts

These initial outcomes suggest that the EMLS could be a useful, parsimonious tool in predicting a range of physical and mental health outcomes. For conditions that are already theorised to be associated with some specific forms of mismatch (e.g., certain types of cancer [20] or attention deficit hyperactivity disorder [22]), this measure will allow researchers to deepen their understanding of this relationship by testing such ideas using a general index of mismatch that contains meaningful subscales, thus revealing what types of mismatches do and do not predict relevant health outcomes. The EMLS, in this regard, could serve as an all-embracing tool (which is backed by a comprehensive framework that could link all the different health-related factors together) in assessing the varying relevance of different health-related factors. For those conditions which have not previously been discussed from a mismatch perspective, the EMLS could provide a quick and efficient way to explore global and specific mismatch associations and such examinations could lead to novel preventative insights as to which modifiable lifestyle and environmental factors might be instrumental in positive health outcomes. For instance, a healthy diet, regular exercise, a dense social network, easy access to nature, and limited social media use is associated with better overall health as reported by participants in our studies.

The current findings have indicated that the scale is reliable (as evidenced by tests of internal consistency, although we have no data yet on test-retest reliability), and has discriminant validity (as demonstrated by its weak-to-non-existent relationships with both the urbanicity and tolerance of ambiguity scales), convergent validity (as shown by its moderately strong relationship with the healthy lifestyle measure), and predictive validity (as evidenced by its ability to predict scores on general physical health issues, general mental wellbeing and overall subjective health).

The predictive validity of the EMLS with regards to all the different health-related measures that were utilised in this project offer broad support for the proposition that evolutionary mismatch has an adverse impact on both physical and mental health. It thus supports a variety of theoretical formulations on how evolutionary mismatch accounts for both physical (e.g. Refs. [17,19,[20], [56]], and mental health issues (e.g., Refs. [21,22,25,57]) that have been largely untested so far. Additional research work can be conducted to evaluate if the EMLS similarly predicts some of these specific health-related conditions (and other currently-unexplored ones), and whether it could likewise be utilised as a quick screening tool in primary care settings either for primary preventive efforts or for early interventional purposes.

### 2.27 Limitations and future directions

As with all research nonetheless, the development of this scale should also be viewed while taking into consideration the limitations of the project. Specifically, as a result of resource restrictions, it should be emphasised that this project has only been conducted with the involvement of participants who were based in the UK and hence the current findings should be interpreted with some degree of caution - in other words, the EMLS was only developed by means of English-speaking, Western, Educated, Industrialised, Rich, and Democratic (WEIRD) samples and so it may potentially not be applicable to other non-English, non-WEIRD societies. It is anticipated that future studies will examine if the 36-item measure is equally applicable to individuals in other parts of the globe, especially those inhabiting non-English speaking and/or non-Western nations, in order to obtain a more complete picture of its utility [58]. The broad hypothesis is that individuals in so-called English-speaking, WEIRD-countries will score higher on various aspects of the EMLS than people in non-English speaking and non-WEIRD countries, although there may be differences between subscales. Furthermore, we hypothesize that people in countries that have undergone rapid cultural and economic changes towards modernity will likewise experience greater EMLS (e.g., countries that have more recently become economically prosperous such as China or India) and may thus report greater health and wellbeing issues. Such propositions should be investigated in future studies.

In addition, as indicated above, participants in the pilot study did not actually appear to agree that most of the proposed items were suitable reflections of evolutionary mismatch as a concept. Nonetheless, we would reiterate our explanation here that the participants in the pilot study have actually regarded not just some, but virtually all of the proposed items (e.g., 88 out of 89 of them) as being unsuitable. While we have no reason to believe that the participants were not fully engaged with the study (particularly when we have already highlighted to the participants the value of being attentive while participating in the study at the outset based on Bayram's [45] approach), it is likely that they might have struggled (especially if they were individuals in the general population without much training in evolutionary behavioural sciences) with understanding what evolutionary mismatch was really about given the abstractness of the concept. This is even more plausible because the proposed items were developed with the help of experts in the field and were based on current theoretical and empirical knowledge of evolutionary mismatch and so should generally have been valid. Notwithstanding, future studies could further evaluate if the scale is valid in other countries/cultures using only evolutionary-minded researchers who are not involved in the project as assessors instead, in order to mitigate the potential issue with understanding what evolutionary mismatch is about.

Furthermore, due to the absence of longer-term data from longitudinal studies in this project (similarly because of resource restrictions), it is not possible to determine conclusively if evolutionary mismatch is indeed influential in ultimately causing poor health outcomes. Future studies exploring the causal influence of evolutionary mismatch on health by means of this scale should strive to adopt a longitudinal or an experimental approach in order to ascertain cause-and-effect relationships. Moreover, such investigations could potentially be enhanced even further with the introduction of certain technologically aided measurements of health (e.g., fitness watches/trackers, glucometers/blood pressure monitors and mental health tracking apps), in addition to the relevant psychometric scales that would typically be adopted.

## 3 Conclusion

Across three studies involving more than 1900 participants, we developed the EMLS, a novel, reliable, and valid 36-item tool that measures individual differences in terms of how evolutionary mismatched people's lifestyles are across various evolutionarily-relevant behavioural lifestyle domains such as diet, exercise, mating, and social connectedness. The EMLS is not only predictive of a range of health-related outcomes (e.g., mental wellbeing, physical health issues, and subjective health) but holds the potential to deepen our understanding of the pre-existing, theorised associations between mismatch and health outcomes while also providing an efficient and easy method of identifying whether mismatch is a contributing factor in other health outcomes hitherto unconceived from this perspective. Mapping individuals in potential risk groups (e.g., children, migrants, and those of lower SES), or even societies or countries that are more at risk, could likewise be an important endeavour in the prevention of a variety of physical diseases (e.g., diabetes) and mental health issues (e.g., depression).

## Data accessibility

Participants did not provide consent to share data publicly. Please do contact the corresponding author if you require more information about the data.

## Ethics and consent section

Ethical approval for the pilot study and all the three main studies were obtained from Aberystwyth University's Department of Psychology ethics committee (AU online ethics number: 10720) on the March 20, 2019, prior to the start of the project. Additional ethical approval was subsequently also obtained for part of the research work from Swansea University's Department of Psychology Ethics Committee (ethics number: 1539) on the October 14, 2019. Informed consent was also obtained from all participants – they were asked to provide their consent by selecting “I agree” after reading the Participant Information and Informed Consent section of the online questionnaire in order to proceed with each study.

## Funding

This project is funded by the Joy Welch Educational Charitable Trust grant awarded to the first author and an Alexander von Humboldt foundation career award to the last author.

## CRediT authorship contribution statement

**Jiaqing O:** Writing – review & editing, Writing – original draft, Resources, Project administration, Methodology, Investigation, Funding acquisition, Data curation, Conceptualization. **Trefor Aspden:** Writing – review & editing, Writing – original draft, Visualization, Validation, Resources, Formal analysis. **Andrew G. Thomas:** Writing – review & editing, Resources. **Lei Chang:** Writing – review & editing, Conceptualization. **Moon-Ho Ringo Ho:** Writing – review & editing, Methodology. **Norman P. Li:** Writing – review & editing, Resources. **Mark van Vugt:** Writing – review & editing, Writing – original draft, Supervision, Funding acquisition, Conceptualization.

## Declaration of competing interest

The authors declare the following financial interests/personal relationships which may be considered as potential competing interests:Mark van Vugt reports financial support was provided by 10.13039/100005156Alexander von Humboldt Foundation. Jiaqing O reports financial support was provided by Joy Welch Educational Charitable Trust.

## Footnotes

Appendix A Supplementary data to this article can be found online at https://doi.org/10.1016/j.heliyon.2024.e34997.

## Footnotes

1 Specifically, participants were told: “The following is a list of statements that we have proposed for a scale on **evolutionary mismatch** (which is a concept that simply means that our physical and mental attributes, which require numerous generations to evolve, might not be perfectly suited for the modernized world and this disparity might lead to some wellbeing issues; in other words, this concept is relating to the question “how natural or unnatural is a particular environment?”). We need your kind assistance in letting us know if these proposed statements are suitable for this scale to the best of your ability. Thank you!”.

2 “Warwick-Edinburgh Mental Well-Being Scale (WEMWBS) © University of Warwick 2006, all rights reserved.”
