# Supplementary material for: Mind the gap: Development and validation of an evolutionary mismatched lifestyle scale and its impact on health and wellbeing
Source: Heliyon. 2024 Jul 23;10(15):e34997. doi: 10.1016/j.heliyon.2024.e34997 (PMC11334630; doi:10.1016/j.heliyon.2024.e34997)
Supplement: Multimedia component 1 [file mmc1.docx]

**Final 36-item Evolutionary Mismatch Scale**

**The following is a list of statements that may be applicable to you (or not). Please try to answer them as honestly as you can.**

1 - Strongly disagree; 2 – Disagree; 3; - Slightly disagree; 4 - Neither agree nor disagree; 5 - Slightly agree; 6 – Agree; 7 - Strongly Agree

1) I live in an area that is densely populated with lots of strangers.

2) I am exposed to very little amount of nature in my home environment.

3) I am not very close to a lot of people in my workplace/place of study (If you are not employed or studying then it will be the place in which you spend most of your day).

4) I send a lot of texts every day.

5) I am generally not very physically active.

6) I do not exercise regularly.

7) I typically spend a large part of my leisure time sat on a chair or sofa.

8) I spend a large part of my life living alone.

9) On average, I get less than half an hour of exercise per day.

10) I have very limited interaction with most of the people in my neighbourhood.

11) I do not actually know most of the people in my neighbourhood.

12) I do not actually know most of my immediate neighbours.

13) I have very limited interaction with most of my relatives.

14) I am emotionally connected to very few of my family members and relatives.

15) I have very few family members and relatives who I could count on if I had big problems.

16) I frequently find that in order to get into an intimate relationship, I have to meet new people and flirt with them.

17) I spend a fair amount of time using apps or dating websites to hook up or have sex.

18) I spend a fair amount of time using apps or dating websites to look at or find potential romantic partners.

19) I feel that in general, I have to rely mostly, if not completely, on my own efforts if I want to get into a romantic relationship with someone.

20) I tend to regularly compare my life with those of other people on the social media.

21) I tend to regularly compare my appearance with those of other people on the social media.

22) I have a lot of “friends” on social media who are mere acquaintances that I don’t know too well.

23) My friends generally use their electronic gadgets/phones/computers most of the time when I am with them.

24) I have very few, if any, close friends who I could count on if I had big problems.

25) I have very few, if any, close friends who I can talk to about my problems and deep secrets.

26) A large part of my diet generally consists of processed meat/vegetables such as those that have been cured, pickled or salted (e.g., lunch meats).

27) I do generally drink a lot of sweetened beverages.

28) A large part of my diet generally consists of food high in fat content.

29) I eat a lot of sugary stuff.

30) I generally do not eat much fresh fruits, vegetables and nuts.

31) I usually sleep alone.

32) I frequently use a lot of different beauty/grooming products to enhance my appearance.

33) I usually spend a considerable amount of time looking at myself in the mirror.

34) I usually spend a considerable amount of time looking at images/videos of myself.

35) I spend a lot of time on Facebook, Twitter, or other social media.

36) I am generally not listened to at my workplace/place of study.
