# Supplementary material for: Mind the gap: Development and validation of an evolutionary mismatched lifestyle scale and its impact on health and wellbeing
Source: Heliyon. 2024 Jul 23;10(15):e34997. doi: 10.1016/j.heliyon.2024.e34997 (PMC11334630; doi:10.1016/j.heliyon.2024.e34997)
Supplement: Multimedia component 2 [file mmc2.pdf]

Gmail Jiaqing O <ojthehuman@gmail.com>

Re: Regarding Paper: The Evolutionary Mismatch Hypothesis: Implications for Psychological Science

11 messages

Becca White <bwhite@psychologicalscience.org> 23 October 2023 at 21:13
To: Jiaqing O <ojthehuman@gmail.com>

Dear OJ,

I can confirm that Heliyon is published by Elsevier, another STM journal, so you may reuse a figure published in Current Directions in your article for Heliyon. Thank you.

Best,

Becca

Becca White

Peer Review Manager
Pronouns: she, her, hers

ASSOCIATION FOR PSYCHOLOGICAL SCIENCE
bwhite@psychologicalscience.org | 202.293.9300
psychologicalscience.org

From: Jiaqing O <ojthehuman@gmail.com>
Sent: Friday, October 20, 2023 1:43 PM
To: Editorial Office <editorialoffice@psychologicalscience.org>
Subject: Fwd: Regarding Paper: The Evolutionary Mismatch Hypothesis: Implications for Psychological Science

Hi there,

Hope this email finds you well. I have emailed a couple of your colleagues about my query below (please kindly see email thread below) but I thought I should send in an email to the general email too just in case. Hope someone is able to answer my query soon if possible as my team are keeping our fingers crossed we will hopefully be able to publish the work soon once we get the clearance for the figure. Thank you so much for your kind assistance in advance - really appreciate it!

Kind Regards
OJ
Assistant Professor
Singapore Institute of Technology

---------- Forwarded message ---------
From: **Jiaqing O** <ojthehuman@gmail.com>
Date: Sat, 21 Oct 2023 at 01:36
Subject: RE: Regarding Paper: The Evolutionary Mismatch Hypothesis: Implications for Psychological Science
To: <rgoldsto@indiana.edu>

Hi Prof Goldstone,

Hope this email finds you well. Hope this email finds you well. We corresponded a while ago while I was at another university pertaining to reusing a figure (Fig. 1) from a publication (entitled "The Evolutionary Mismatch Hypothesis: Implications for Psychological Science") in Current Directions in Psychological Science for our paper a while ago (if you remember) and you said it was fine to do so and your colleague, Amy Drew, also indicated that it was fine as our

originally intended journal, Personality and Individual Differences has some sort of agreement with Sage and other journals to reuse materials under the STM agreement between journals. To this end, as we have now been invited to resubmit our paper to another journal (Heliyon) instead, we are just wondering does the same approach apply in this case? That is, if we can freely reuse the figure in our paper if it is published (open access) by just stating the following: "Reused from Li, van Vugt, & Colarelli (2018, p. 40) under the STM agreement"? Or do we need any additional permission or statement to indicate that the figure is not reuseable even if the paper is open access? Thanks for your kind attention once again!

Kind Regards
OJ
Assistant Professor
Singapore Institute of Psychology

Virus-free.www.avast.com

Jiaqing O <ojthehuman@gmail.com> 23 October 2023 at 21:36
To: Becca White <bwhite@psychologicalscience.org>

Hi Becca,

Thanks for your reply. I understand that Heliyon is a STM journal. What I wasn't sure is if we need additional permission for it to appear in Heliyon, which is also an open access journal? More specifically, just wondering what should we write as the copyright statement when reusing it in our paper which will be an open access one and the original paper in Current Directions is not? I have asked a number of people and have even tried to get permission from Sage but none of these produce any clear answer and so it would be greatly appreciated if you could help, thanks!

Kind Regards
OJ
[Quoted text hidden]

Becca White <bwhite@psychologicalscience.org> 23 October 2023 at 22:26
To: Jiaqing O <ojthehuman@gmail.com>

Hi OJ,

You do not need additional permission, and the open access status doesn't matter with regard to how it is cited. For the citation, it should read as follows:

> Li, N. P., van Vugt, M., & Colarelli, S. M. (2018). The Evolutionary Mismatch Hypothesis: Implications for Psychological Science. Current Directions in Psychological Science, 27(1), 38-44. https://doi.org/10.1177/0963721417731378

You do not need to mention the STM agreement in the citation. Please let me know if you have any additional questions.

Best,

Becca

Becca White

Peer Review Manager
Pronouns: she, her, hers

ASSOCIATION FOR PSYCHOLOGICAL SCIENCE
bwhite@psychologicalscience.org | 202.293.9300
psychologicalscience.org

From: Jiaqing O <ojthehuman@gmail.com>
Sent: Monday, October 23, 2023 9:36 AM
To: Becca White <bwhite@psychologicalscience.org>
Subject: Re: Regarding Paper: The Evolutionary Mismatch Hypothesis: Implications for Psychological Science

[Quoted text hidden]

Jiaqing O <ojthehuman@gmail.com> 24 October 2023 at 01:39
To: Becca White <bwhite@psychologicalscience.org>

Hi Becca,

Right I see - so just wondering what do I write at the bottom of the figure? Do I need to say "reprinted with permission from Li, van Vugt and Colarelli (2018)..." or something like that? In other words, is there like a standard way of phrasing it under the STM agreement?

In addition, I was trying to read up more about it and I found the following:

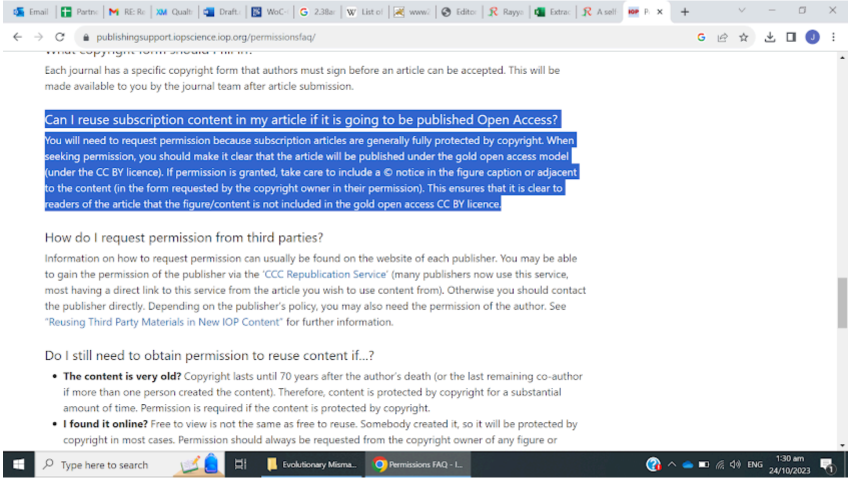

publishingsupport.iopscience.iop.org/permissionsfaq/

Each journal has a specific copyright form that authors must sign before an article can be accepted. This will be made available to you by the journal team after article submission.

Can I reuse subscription content in my article if it is going to be published Open Access?

You will need to request permission because subscription articles are generally fully protected by copyright. When seeking permission, you should make it clear that the article will be published under the gold open access model (under the CC BY licence). If permission is granted, take care to include a © notice in the figure caption or adjacent to the content (in the form requested by the copyright owner in their permission). This ensures that it is clear to readers of the article that the figure/content is not included in the gold open access CC BY licence.

How do I request permission from third parties?

Information on how to request permission can usually be found on the website of each publisher. You may be able to gain the permission of the publisher via the 'CCC Republication Service' (many publishers now use this service, most having a direct link to this service from the article you wish to use content from). Otherwise you should contact the publisher directly. Depending on the publisher's policy, you may also need the permission of the author. See "Reusing Third Party Materials in New IOP Content" for further information.

Do I still need to obtain permission to reuse content if...?

- **The content is very old?** Copyright lasts until 70 years after the author's death (or the last remaining co-author if more than one person created the content). Therefore, content is protected by copyright for a substantial amount of time. Permission is required if the content is protected by copyright.
- **I found it online?** Free to view is not the same as free to reuse. Somebody created it, so it will be protected by copyright in most cases. Permission should always be requested from the copyright owner of any figure or

Type here to search Evolutionary Misma... Permissions FAQ - I... ENG 1:30 am 24/10/2023

The highlighted bits on this website seemed to suggest a specific approach to referencing a diagram from a traditional journal for one that is open access. I wonder if I need to do the same for my case?

Thanks again for your kind assistance. Really appreciate it!
OJ

[Quoted text hidden]

Becca White <bwhite@psychologicalscience.org> 24 October 2023 at 05:23
To: Jiaqing O <ojthehuman@gmail.com>
Cc: Amy Drew <adrew@psychologicalscience.org>

For the citation under the figure, please use the following wording:

Reprinted with permission from Li, N. P., van Vugt, M., & Colarelli, S. M. (2018). The Evolutionary Mismatch Hypothesis: Implications for Psychological Science. *Current Directions in Psychological Science.* Copyright 2018, Association for Psychological Science.

This will make it clear that the figure is copyrighted by APS and not under the CC BY license of your open access article. Thank you!

Best,

Becca

Becca White

Peer Review Manager
Pronouns: she, her, hers

ASSOCIATION FOR PSYCHOLOGICAL SCIENCE
bwhite@psychologicalscience.org | 202.293.9300
psychologicalscience.org

From: Jiaqing O <ojthehuman@gmail.com>
Sent: Monday, October 23, 2023 1:39 PM
[Quoted text hidden]

[Quoted text hidden]

Jiaqing O <ojthehuman@gmail.com> 24 October 2023 at 13:05
To: Becca White <bwhite@psychologicalscience.org>
Cc: Amy Drew <adrew@psychologicalscience.org>

Hi Becca,

Thank you so much for your helpful clarification! If that is the case, may I confirm that all I need to do is just to include that wording you have provided under the figure even if I am trying to publish in an open access journal and I wouldn't need any other kind of permission (or any other thing) from anyone else or to add any other wording as we are still in adherence to the STM agreement? Thanks again!

Kind Regards
OJ
[Quoted text hidden]

Becca White <bwhite@psychologicalscience.org> 24 October 2023 at 22:28
To: Jiaqing O <ojthehuman@gmail.com>
Cc: Amy Drew <adrew@psychologicalscience.org>

Hi OJ,

Yes, I confirm that the wording I provided is all you need, and you do not need anything else with regard to the STM agreement. If Heliyon asks you to provide proof of permission, you can just send or upload this email.

Best,

Becca

Becca White

Peer Review Manager
Pronouns: she, her, hers

ASSOCIATION FOR PSYCHOLOGICAL SCIENCE
bwhite@psychologicalscience.org | 202.293.9300
psychologicalscience.org

From: Jiaqing O <ojthehuman@gmail.com>
Sent: Tuesday, October 24, 2023 1:05 AM
To: Becca White <bwhite@psychologicalscience.org>
Cc: Amy Drew <adrew@psychologicalscience.org>
[Quoted text hidden]

[Quoted text hidden]

Jiaqing O <ojthehuman@gmail.com> 25 October 2023 at 11:19
To: Becca White <bwhite@psychologicalscience.org>
Cc: Amy Drew <adrew@psychologicalscience.org>

Hi Becca,

Great thanks!! Really appreciate it! You have been so helpful with this! Many thanks!

Thanks
OJ
[Quoted text hidden]

Jiaqing O <ojthehuman@gmail.com> 17 June 2024 at 11:37
To: Becca White <bwhite@psychologicalscience.org>

Hi Becca,

Hope this email finds you well. Just wondering if I could double-check with you whether the same wording applies if I am also including the same caption for the figure from the original article? Thanks!

Kind Regards
OJ
[Quoted text hidden]

Becca White <bwhite@psychologicalscience.org> 17 June 2024 at 20:40
To: Jiaqing O <ojthehuman@gmail.com>

Hi OJ,

Yes, the wording is the same if you include the figure caption as well.

Best,

Becca White

Senior Publications Manager
Pronouns: she, her, hers

ASSOCIATION FOR PSYCHOLOGICAL SCIENCE
bwhite@psychologicalscience.org | 202.293.9300
psychologicalscience.org

From: Jiaqing O <ojthehuman@gmail.com>
Sent: Sunday, June 16, 2024 11:37 PM
[Quoted text hidden]

[Quoted text hidden]

Jiaqing O <ojthehuman@gmail.com> 18 June 2024 at 08:13
To: Becca White <bwhite@psychologicalscience.org>

Hi Becca,

Great, thanks a lot for your kind assistance!

Many thanks
OJ
[Quoted text hidden]
